# Mechanical loading of tissue engineered skeletal muscle prevents dexamethasone induced myotube atrophy

**DOI:** 10.1007/s10974-020-09589-0

**Published:** 2020-09-21

**Authors:** Kathryn W. Aguilar-Agon, Andrew J. Capel, Jacob W. Fleming, Darren J. Player, Neil R. W. Martin, Mark P. Lewis

**Affiliations:** 1 School of Sport, Exercise and Health Sciences, Loughborough University, Loughborough, LE11 3TU UK; 2 Division of Surgery and Interventional Science, Faculty of Medical Sciences, University College London, London, UK

**Keywords:** Dexamethasone, C2C12, Hypertrophy, Skeletal muscle, Myotubes, Ubiquitin–proteasome

## Abstract

Skeletal muscle atrophy as a consequence of acute and chronic illness, immobilisation, muscular dystrophies and aging, leads to severe muscle weakness, inactivity and increased mortality. Mechanical loading is thought to be the primary driver for skeletal muscle hypertrophy, however the extent to which mechanical loading can offset muscle catabolism has not been thoroughly explored. In vitro 3D-models of skeletal muscle provide a controllable, high throughput environment and mitigating many of the ethical and methodological constraints present during in vivo experimentation. This work aimed to determine if mechanical loading would offset dexamethasone (DEX) induced skeletal muscle atrophy, in muscle engineered using the C2C12 murine cell line. Mechanical loading successfully offset myotube atrophy and functional degeneration associated with DEX regardless of whether the loading occurred before or after 24 h of DEX treatment. Furthermore, mechanical load prevented increases in MuRF-1 and MAFbx mRNA expression, critical regulators of muscle atrophy. Overall, we demonstrate the application of tissue engineered muscle to study skeletal muscle health and disease, offering great potential for future use to better understand treatment modalities for skeletal muscle atrophy.

**Electronic supplementary material:**

The online version of this article (10.1007/s10974-020-09589-0) contains supplementary material, which is available to authorized users.

## Introduction

Skeletal muscle atrophy is known to occur as a consequence of acute and chronic illnesses (such as sepsis, chronic kidney disease and cancer cachexia), immobilisation or bed rest, muscular dystrophies, and aging. This can lead to severe muscle weakness, inactivity and reduced quality of life for the patients. Whilst the imbalance caused in all common forms of atrophy between protein synthesis and degradation is paramount in the aetiology of muscle atrophy (Jackman and Kandarian 2004; Lecker et al. 2004), the underpinning molecular mechanisms have yet to be fully defined. Nevertheless, under conditions of muscle atrophy the primary degradative pathway in skeletal muscle is the ubiquitin proteasome pathway (UPP). Transcriptional profiling has identified Muscle Atrophy F-box (MAFbx) and Muscle RING-finger protein-1 (MuRF-1) as two muscle-specific ubiquitin ligases, which express relatively low levels under resting conditions but are rapidly upregulated under atrophy-inducing conditions (Bodine et al. 2001; Gomes et al. 2001). Thus, MAFbx and MuRF-1 have been described as crucial regulators of the atrophy process through the degradation of contractile proteins via the UPP. Furthermore, the examination of muscle atrophy in mice containing null deletions of MAFbx (Bodine et al. 2001) or MuRF-1 (Bodine et al. 2001; Gomes et al. 2012; Labeit et al. 2010), has further supported the importance of both these ubiquitin ligases in the regulation of muscle atrophy.

Resistance exercise is well established as a successful treatment for the loss of muscle size and strength. Indeed, resistance exercise is efficacious in being able to maintain or augment muscle volume following bouts of unloading (Schulze et al. 2002; Tesch 2004), such as stimulated spaceflight and ageing (Akima et al. 2001,2000; Hunter et al. 2004)*.* In rodents, compensatory hypertrophy and resistance type exercise has also successfully reversed muscle atrophy (Gardiner et al. 1980; Goldberg and Goodman 1969). Additionally, rodent studies have highlighted the ability of exercise treatments to reduce the magnitude of muscle atrophy when performed prior to immobilization, as well as afterwards (Petrini et al. 2016; Sun et al. 2013)*.* However, the beneficial effects of resistance exercise, both prior to and following an atrophic stimulus, are yet to be fully elucidated. Consequently the optimal frequency, intensity, and timing of muscle loading to perform under atrophy-inducing conditions to fully restore skeletal muscle mass and function are unknown. Moreover, delineating the mechanisms regulating muscle size under these conditions of atrophy and hypertrophy are difficult to explore using in vivo models, due to the inherent difficulties associated with modelling complex multi-facetted biological systems.

In vitro systems can produce some insights into skeletal muscle adaptation under situations of muscle atrophy and loss of strength. In particular, 3D tissue engineered skeletal muscle systems are advantageous since they accurately recapitulate the architecture and function of native skeletal muscle tissue (Kasper et al. 2018; Khodabukus et al. 2007).Certainly, it is possible to produce an exercise-like stimulus to 3D skeletal muscle tissue in vitro via the delivery of electrical pulse stimulation or mechanical loading (Khodabukus and Baar 2012; Sasai et al. 2010; Vandenburgh et al. 1989; Vandenburgh and Kaufman 1979) Recently we have published the positive effects of mechanical loading tissue engineered skeletal muscles on the ubiquitin ligase MAFbx (Aguilar‐Agon et al. 2019). Mechanical loading resulted in large increases in myotube width and muscle functionality, alongside increases in IGF-1 mRNA expression and increased phosphorylation of mTORC1 targets. This response successfully replicated the effects commonly seen following resistance exercise in humans (Bamman et al. 2001; Louis et al. 2007). Therefore, through these well-known mechanical signalling pathways, mechanical loading is thought to be the primary driver for hypertrophy. Hence, mechanical load of tissue engineered skeletal muscle in vitro has allowed us to examine and induce skeletal muscle hypertrophy at a molecular, morphological and functional level (Aguilar‐Agon et al. 2019; Player et al. 2014). However, it is not well known whether mechanical loading of engineered muscle is able to protect myotubes from an atrophic stimulus, thus replicating in vivo physiology, and therefore more investigations are required to validate these in vitro systems prior to their future use in the field.

In the present study we aimed to determine the beneficial effects of mechanical loading on engineered muscle both prior to and following an atrophic stimulus. To achieve myotube atrophy we used the synthetic glucocorticoid (GC) dexamethasone (DEX). DEX has successfully been used for inducing muscle atrophy in both monolayer skeletal muscle cells and 3D engineered muscle in vitro (Barassi et al. 2016; Castillero et al. 2013; Shimizu et al. 2017)*.* GCs are a class of steroid hormones secreted by the adrenal glands and are potent mediators of muscle wasting in many catabolic conditions e.g. sepsis, cachexia, starvation, metabolic acidosis and severe insulinopenia (Goldberg et al. 1980), initiating protein degradation in part by increasing the expression of several components of the UPP, including MAFbx and MuRF-1, (Bodine et al. 2001); or direct degradation of proteins by the proteasome (Mitch and Goldberg 1996). We hypothesized that mechanical loading before and after DEX treatment will successfully alleviate the detrimental effects of DEX on catabolic gene expression, myotube atrophy and functionality of the engineered muscles.

## Methods

### Cell culture

The immortalised C2C12 murine skeletal muscle myoblast cell line (ECACC, Sigma Aldrich, UK) was used for all experiments described. C2C12′s were sub-cultured in T80 flasks (Nunc, Fisher Scientific, UK) in growth media (GM: high glucose Dulbecco’s modified Eagle´s media (DMEM, Fisher)), supplemented with 20% (v/v) fetal bovine serum (FBS, PAN Biotech, Germany) and 1% (v/v) penicillin–streptomycin (P/S, Gibco, UK), then incubated in a humidified 5% CO_2_ atmosphere at 37 °C. GM was changed daily until 80% confluence was reached. For all experimentation cells had undergone fewer than 11 passages.

### Fabrication of tissue engineering skeletal muscle

The printing and modelling of the tissue engineering moulds was performed as described in (Aguilar‐Agon et al. 2019; Capel et al. 2019). All the relevant standard tessellation language (.stl) files for the design explained within this manuscript are available to download at the following domain: https://figshare.com/projects/3D_Printed_Tissue_Engineering_Scaffolds/36494. Printed inserts were adhered to culture well plates using polydimethylsiloxane (PDMS, Sylgard® 184, Dow Corning), sterilised with 70% industrial methylated spirits (IMS, Fisher) and left to evaporate under UV irradiation for 24 h prior to use. Briefly, 10% (v/v) 10 × minimum essential medium (MEM, Gibco) was added to 65% type I rat-tail collagen (First Link, UK.; in 0.1 M acetic acid, protein concentration 2.035 mg/mL). The solution was neutralized using 5 M and 1 M sodium hydroxide (NaOH) in a drop-wise fashion until an observed colour change (yellow to cirrus pink). Subsequently, 4 × 10^6^ cells/mL C2C12s were added to 5% DMEM and 20% v/v Corning ® Matrigel® Matrix (Corning, Germany). The final solution was pipetted into each 3D printed custom well mould. The engineered muscles were then placed in a 37 °C humidified incubator with 5% CO_2_ for up to 20 min to set. 3 mL of GM was added to each engineered muscle and incubated for 4 days, with media replenished daily. GM was removed and replaced with differentiation media (DM: high glucose DMEM (Sigma)), supplemented with 2% horse serum (Sigma) and 1% P/S (Gibco)), which was changed daily for a further 10 days.

### Treatment with dexamethasone and mechanical load

DEX (Sigma) was used to induce atrophy in the 3D tissue engineered skeletal muscle. Tissue engineered skeletal muscles were serum starved for 4 h prior to DEX treatment (DMEM and 1% PS). DEX was diluted in ethanol (Sigma) to a concentration of 12.5 mg/mL and mixed with DM. Preliminary experiments were carried out whereby the engineered muscles were treated with varying doses of DEX (10, 20, 40, 80 and 100 µM) to ascertain the optimal dose for inducing muscle atrophy without cell death. On day 14, following 4 h of serum starvation, tissue engineered muscles were treated with DEX by medium alteration and cultured for 24 h. Prior to or following DEX treatment selected engineered muscles were floated in DM and progressively mechanically loaded using a mechanical stimulation bioreactor (MSB). This progressive load regime, as previously described (Aguilar‐Agon et al. 2019), consists of a continuous increasing load to achieve 15% stretch over a 1 h period, with the engineered muscle left thereafter under tension (15% stretch) for a further 2 h, enabling maximal mechanical load to be placed upon the myotubes. Upon the cessation of experiments, constructs were either fixed in 3.75% formaldehyde solution (Sigma) for immunohistochemistry or homogenized in TRIzol™ Reagent (Invitrogen, Fisher) to extract RNA.

### RNA extraction and RT-qPCR

Following the cessation of experimentation engineered muscles were homogenised in 500 µL of TRizol™ Reagent using TissueLyser beads (Qiagen, UK) and disrupted for 3 × 120 s at 20 Hz using the TissueLyser II (Qiagen). RNA extraction was performed using the TRIzol method as described in the manufacturer’s instructions. RNA quality and concentration were measured by UV spectroscopy at optical densities of 260 and 280 nm using a Nanodrop 2000 spectrophotometer (ThermoFisher Scientific, USA). One-step RT-PCR amplifications were carried out using Quantifast SYBR Green RNA-to-CT 1 step kit (Qiagen), loading 20 ng of RNA per reaction on a ViiA7™ Real-Time PCR System (Applied Bio-system, ThermoFisher), and analysed using ViiA7™ RUO software. RT-PCR procedure was as follows: 50 °C, 10 min (for cDNA synthesis), 95 °C, 5 min (transcriptase inactivation), followed by 95 °C, 10 s (denaturation), 60 °C, 30 s (annealing/extension) for 40 cycles. Melt curve analyses were performed to determine and omit non-specific amplification or primer-dimer samples. Relative gene expressions were calculated using the comparative CT (Δ^ΔCt^) equation for normalised expression ratios (Schmittgen and Livak 2008); relative expression calculated as 2^-ΔΔCt^, where C_t_ is representative of the threshold cycle. POLR2B (RPII β) was used as the housekeeping gene in all RT-PCR assays, and data was calibrated to a single control (CON) sample from each independent experiment. Primer information can be found in Table 1. All reactions were performed in triplicate.

**Table 1:** Primer sequences for the housekeeping gene POLR2B and the E3 ubiquitin ligases MuRF-1 and MAFbx which were used as markers of muscle atrophy

	Forward (5′-3′)	Reverse (5′-3′)	Product length	NCBI reference sequence
POLR2B	GGTCAGAAGGGAACTTGTG	GCATCATTAAATGGAGTAG	148	NM_153798.2
MAFbx	GTCGCAGCCAAGAAGAGAA	CGAGAAGTCCAGTCTGTTGAA	134	NM_026346.3
MuRF-1	AAACAGGAGTGCTCCAGTCGG	CGCCACCAGCATGGAGATACA	67	NM_001039048.2

*POLR2B* RNA polymerase II beta, *MuRF-1* muscle ring finger protein-1, *MAFbx* muscle atrophy F box

### Histology

Fixed engineered muscles were dehydrated in 20% sucrose (v/v in tris-buffered saline (TBS)) for 24 h. Engineered muscles were then embedded in Tissue-Tek ® (VWR, USA) optimum cutting temperature (O.C.T) mounting medium and frozen at − 80 °C. Once frozen, serial transverse cross-section (12 µm) from the mid-belly of each 3D tissue engineered skeletal muscle were cut at − 20 °C using standard cryostat protocols, perpendicular to the longitudinal axis of the engineered muscle. All sections were placed on poly-lysine microscope slides (Fisher) and stored at -70 °C. For Immunohistochemical analysis, frozen serial muscle sections were thawed and air-dried at RT. Sections were then permeabilised and blocked with a solution containing 5% goat serum (GS, Fisher), 0.2% Triton-X100 (Sigma) and TBS at RT for 30 min. Samples were then incubated at RT overnight with the MyHC antibody MF-20, which was deposited to the DSHB by Fischman, D.A (1:200, DSHB Hybridoma Product MF-20). Then replaced with the secondary anti-body Alexa Fluor™ 488 goat anti-mouse (1:500, Invitrogen) and 4′,6-diamidino-2-phenylindole (DAPI,1:2000) in blocking solution for 2 h in the dark at RT. Thereafter slides were washed 3 × TBS and sealed with glass cover slides (22 × 50 mm, Menzel, UK) using Fluoromount™ aqueous mounting medium (Sigma). Images were captured using a Leica DM2500 fluorescent microscope at 10 × magnifications and morphological analysis was carried out using the image processing package FIJI (ImageJ, SciJava), with a minimum of 5 images analysed per engineered muscle. Within each image average myotube cross-sectional area (CSA), average myotube width and total MyHC positive cells per mm^2^ were calculated using an in-house macro implemented using FIJI (supplementary material).

### Assessment of functional muscle

To determine if mechanical loading had protected or reversed DEX-induced decreases in maximal tetanic skeletal muscle force, engineered muscles were immersed in 3 mL Krebs–Ringer-HEPES buffer (KRH; 10 mM HEPES, 138 mM NaCl, 4.7 mM KCl, 1.25 mM CaCl_2_, 1.25 mM MgSO_4_, 5 mM Glucose, 0.05% bovine serum albumin in dH_2_0) and attached to a force transducer (403A, Auora Scientific Ltd, UK). Wire electrodes were positioned parallel to the engineered muscles to allow for electric field stimulation. Impulses were generated using LabVIEW software (National Instruments, United Kingdom) connected to a custom-built amplifier. Maximal tetanic force was determined using a single 3.6 V/mm, 1.2 ms impulse and maximal tetanic force was measured using a 1 s pulse train at 100 Hz and 3.6 V/mm, generated using LabVIEW 2012 software (National Instruments). Tetanus data was derived from three contractions per construct. Data was acquired using a Powerlab system (ver. 8/35) and associated software (Labchart 8, AD Instruments, UK).

### Statistical analysis

All statistical analysis was performed using SPSS software version 23 (SPS INC, USA). Normality of distribution and homogeneity of variance in all data sets were determined using a Shapiro–Wilk test and Levine’s test, respectively. Data sets were then appropriately analysed using a One-Way ANOVA with an LSD post-hoc test. A Kruskal–Willis test was performed where data was not normally distributed. All data is presented as mean ± standard deviation (SD).

## Results

### Addition of DEX successfully decreases average myotube cross sectional area and force production

**Fig. 1:**
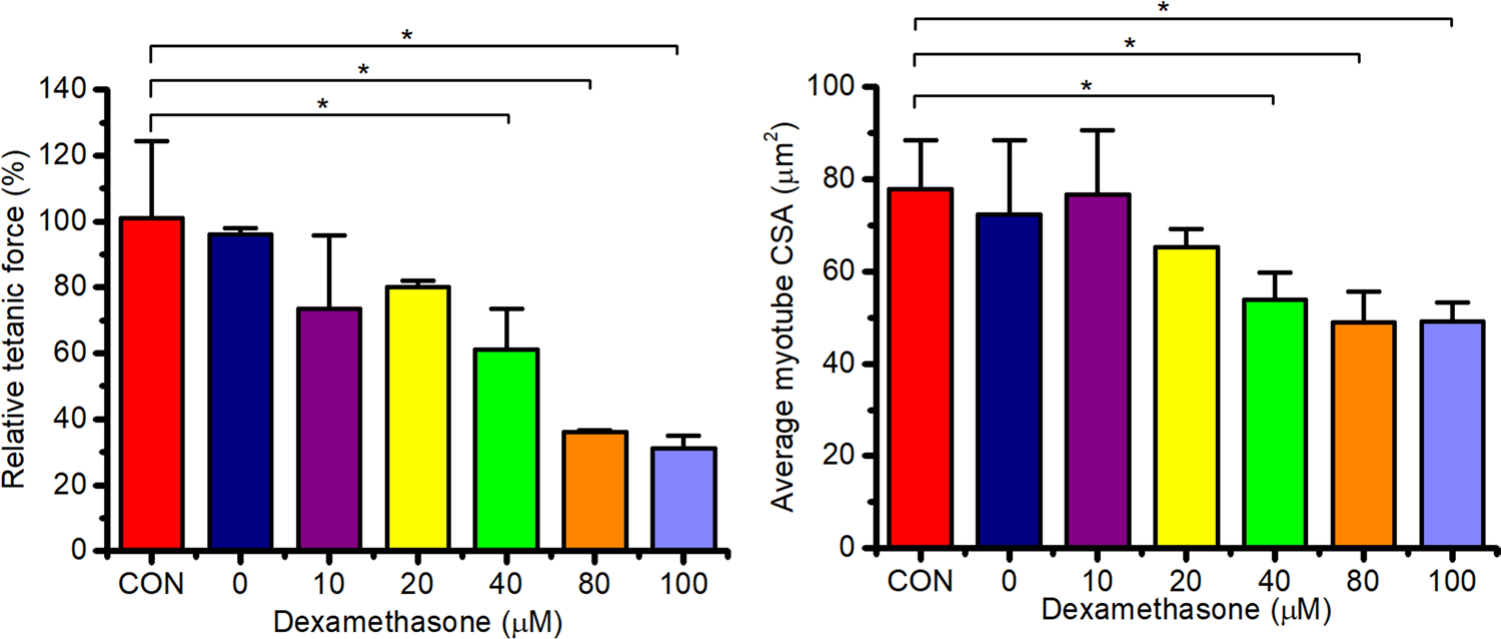
Loss of contractile force and myotube cross sectional area (CSA) (µm^2^) of 50 µl engineered skeletal muscle cultures 24 h post DEX treatment (0, 10, 20, 40, 80 and 100 µM). All cultures were compared to no DEX controls (CON) at day 14 within individual experimental repeats to calculate relative force. Significant values are identified using * where a significance of P ≤ 0.05 was achieved. All data presented as mean ± SD, from n = 5 engineered muscles, obtained from three independent experiments

To determine an appropriate dose of DEX administration to produce significant atrophy of the engineered muscles, we administered doses of DEX ranging from 0 to 100 µM over 24 h. Maximal tetanic force output and average cross-sectional area (CSA) of the myotubes in the engineered skeletal muscles was measured after 24 h (Fig. 1). Contractile force was compared to that of control engineered muscle, in which DEX was not administered (CON). Contractile force significantly decreased in a dose-dependent manner as DEX was increased above 20 µM (P < 0.05) compared to CON, plateauing at 80 µM. Maximal force production was reduced significantly by ~ 40%, 65% and 70% following 40, 80 and 100 µM of DEX administration respectively compared to CON. Furthermore, it was also determined that loss of myotube CSA appeared to occur in a dose-dependent fashion, with the higher doses (> 40 µM) of DEX associated with significant atrophy (P < 0.05), compared to CON muscle, with myotube CSA decreased by ~ 31–37% at 40–100 µM of DEX. This in part could explain the loss of maximal tetanic force production following DEX treatment. No differences were observed in contractile force and myotube CSA when comparing the vehicle controls (0 µM: addition of the relative ethanol concentration administered at the highest dose of DEX) and CON. Thus, 40 µM of DEX administration was chosen for further experimentation as it was the lowest concentration to result in significant atrophy of the myotubes and loss of functionality within the engineered muscles.

### Mechanical loading induces a downregulation in ubiquitin ligases MAFbx and MuRF-1 mRNA expression following dexamethasone administration

**Fig. 2:**
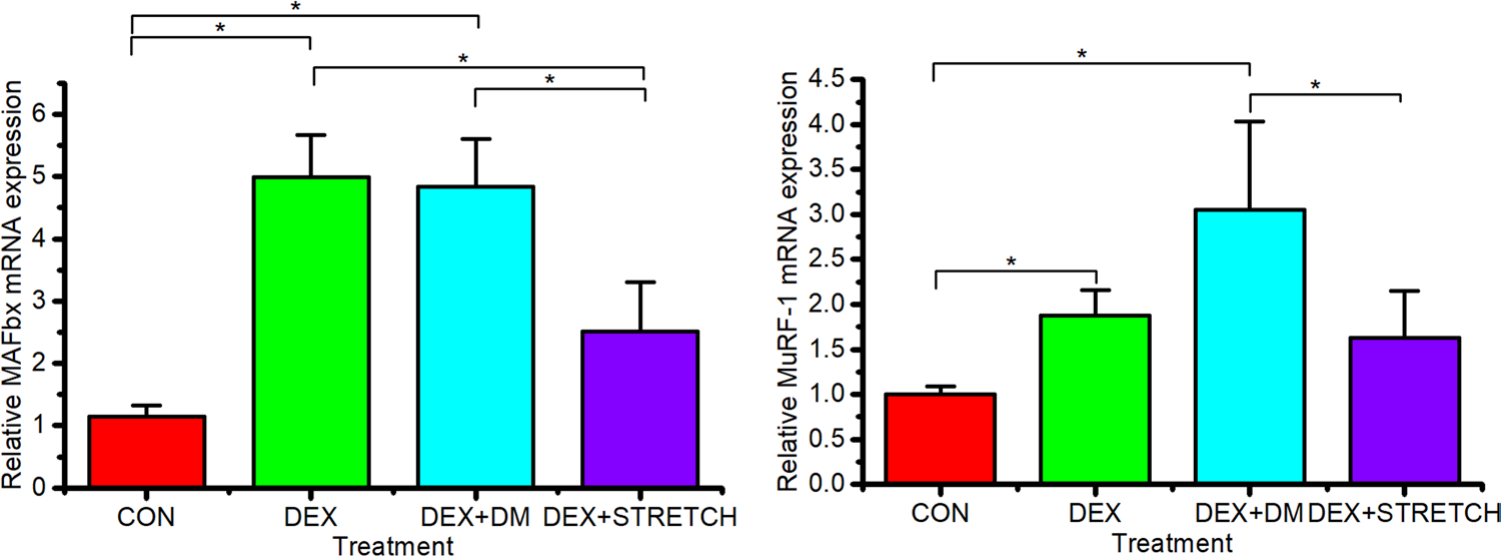
MAFbx and MuRF-1 ΔΔCT expression level of CON(control day 14, n = 6), 40 µM DEX administration over 24 h (DEX, n = 11), 40 µM DEX administration over 24 h replaced with differentiation media for 45 h (DEX + DM, n = 10) and 40 µM DEX administration over 24 h replaced with differentiation media and mechanically loaded for 3 h and sampled after 45 h (DEX + STRETCH, n = 11). Significant values are identified using * where a significance of P ≤ 0.05 was achieved. All data presented as mean ± SD, obtained from three independent experiments

Due to the importance of the UPP in protein degradation, mRNA levels of ubiquitin ligases MAFbx and MuRF-1 were investigated following DEX-induced atrophy ± mechanical loading (Fig. 2). 40 µM of DEX was administered to the tissue engineered muscles for 24 h to induce atrophy. Subsequently, engineered muscles were either incubated for 48 h in differentiation media or mechanically loaded in differentiation media for 3 h and then incubated for 45 h at resting length. Both MAFbx and MuRF-1 mRNA expression were significantly upregulated following 24 h of DEX administration compared to CON (P < 0.05). In contrast, the upregulation of MAFbx and MuRF-1 mRNA expression following DEX administration was significantly reduced following a bout of mechanical load compared to DEX (P < 0.05). No significant differences were observed between MAFbx and MuRF-1 mRNA expression following mechanical load post DEX administration vs CON engineered muscles (p > 0.05), suggesting mechanical loading is sufficient in restoring control level expression. Moreover, the addition of differentiation media over a 48 h period following an acute bout of DEX administration did not significantly reduce MAFbx and MuRF-1 mRNA expression when compared to 24 h of DEX administration (P > 0.05). Interestingly, MuRF-1 mRNA expression peaked 48 h following the removal of DEX, describing a ~ threefold increase in MuRF-1 mRNA expression levels (P < 0.05). Overall, mechanical loading was necessary to reverse the continuing atrophic effects induced through DEX administration on ubiquitin ligases MAFbx and MuRF-1.

### Mechanical loading recovers DEX-induced myotube atrophy

**Fig. 3:**
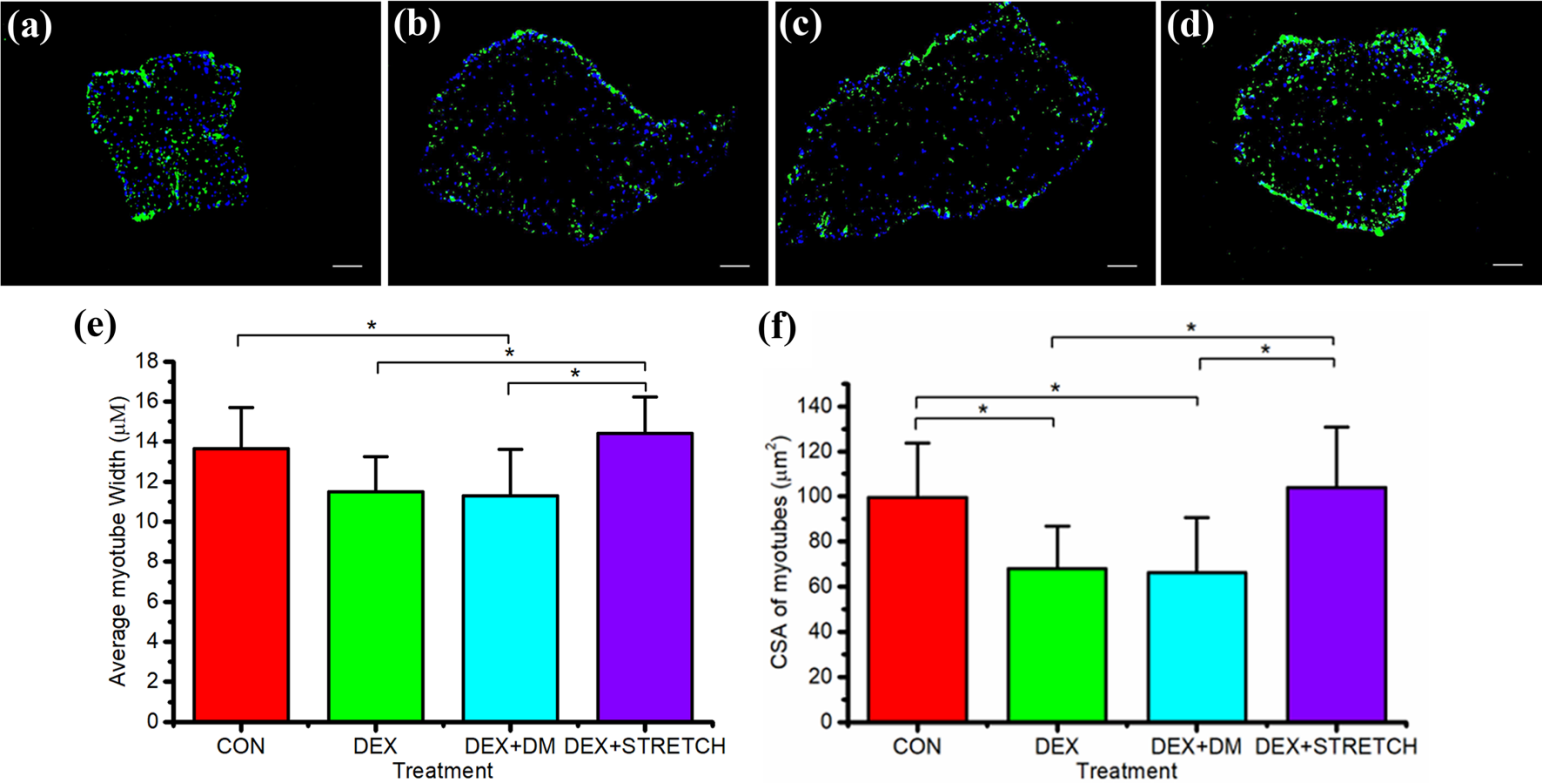
Immunohistochemical fluorescent staining of the nucleic DNA (blue) and muscle specific protein filament MyHC (green) in cross sections of engineered muscles (× 10 magnification) **a** representative CON (no DEX administration at day 14, n = 7), **b** 40 µM DEX administration over 24 h (DEX, n = 8), **c** 40 µM DEX administration over 24 h replaced with differentiation media for 48 h (DEX + DM, n = 7), **d** 40 µM DEX administration over 24 h replaced with differentiation media and mechanically loaded for 3 h and sampled after 48 h (DEX + STRETCH, n = 6), **a** average myotube width (µm), **f** average cross sectional area (CSA) of the myotubes (μm^2^) of CON (Control at day 14), DEX, DEX + DM and DEX + STRETCH. Scale bar represents 100 μm. Significantly different values are identified using*. All data presented as mean ± SD, obtained from three independent experiments. (Color figure online)

Following evidence of the action of members of the UPP, we sought to examine whether this translated into an atrophic effect. Next, we examined whether mechanical load could reverse the significant loss in average myotube width and CSA following 24 h of DEX administration (Fig. 3). Mechanical load of the engineered muscles post DEX administration restored both the average CSA and width of the myotubes compared to DEX administration alone (P = 0.005 and 0.009). No significant differences in myotube width and CSA were observed when comparing mechanical loading post DEX administration and CON (P = 0.489 and 0.724 respectively). Moreover, the addition of differentiation media post DEX administration did not reverse myotube atrophy alone (width: P = 0.997 and CSA: P = 0.947), demonstrating that this effect was specific to the application of mechanical loading and not time in culture.

### Mechanical loading prior to DEX administration prevents elevations in MAFbx and MuRF-1 mRNA and myotube atrophy

**Fig. 4:**
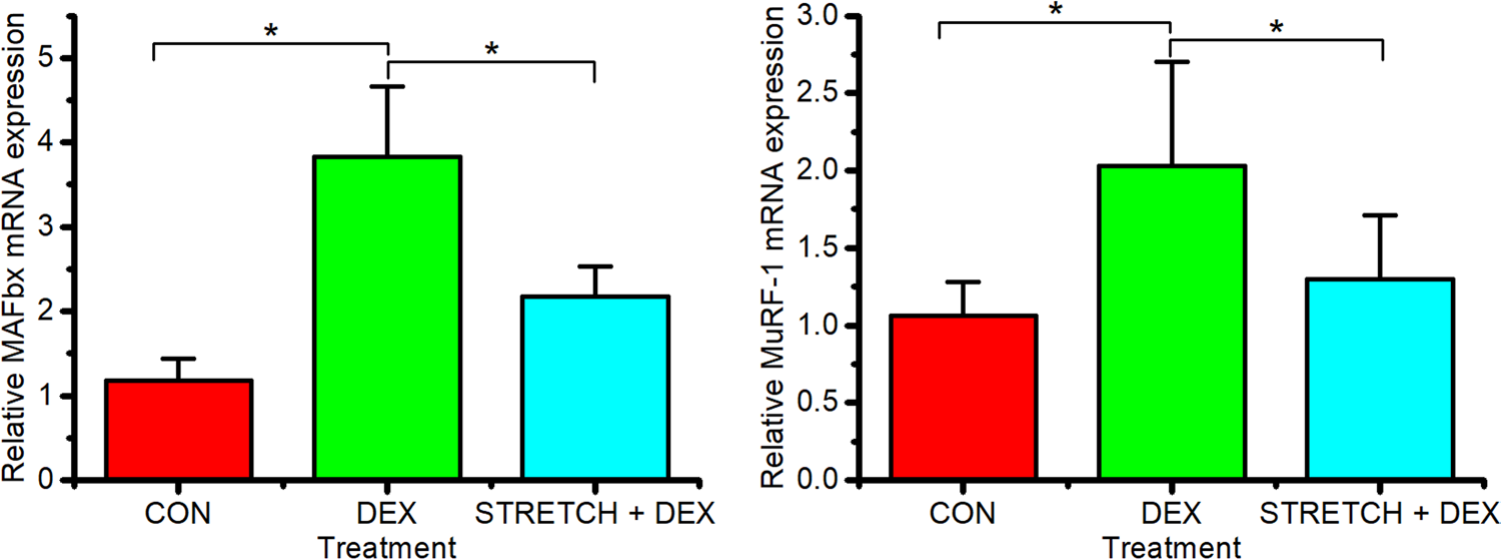
MAFbx and MuRF-1 ΔΔCT expression level of CON (Control day 14, n = 5), 40 µM DEX administration over 24 h (DEX, n = 12), and mechanically loaded for 3 h then administered 40 µM DEX over 24 h (STRETCH + DEX, n = 9). Significant values are identified using * where a significance of P ≤ 0.05 was achieved. All data presented as mean ± SD, obtained from three independent experiments

Following the success of mechanical load at preventing DEX induced catabolic mRNA expression and atrophy, we next tested the hypothesis that mechanical load prior to the administration of DEX would have a protective effect on ubiquitin ligases MAFbx and MuRF-1 and prevent DEX induced atrophy. We observed that mechanical loading prior to DEX administration, prevented the induction of both MAFbx and MuRF-1 that is observed with DEX alone (DEX administration over 24 h, P < 0.05, Fig. 4). Indeed, compared to control samples, MAFbx and MuRF-1 mRNA levels remained unchanged when mechanical loading preceded DEX administration (P = 0.998 and P = 0.286 respectively), providing evidence of a protective effect of mechanical loading on molecular regulators of muscle atrophy.

**Fig. 5:**
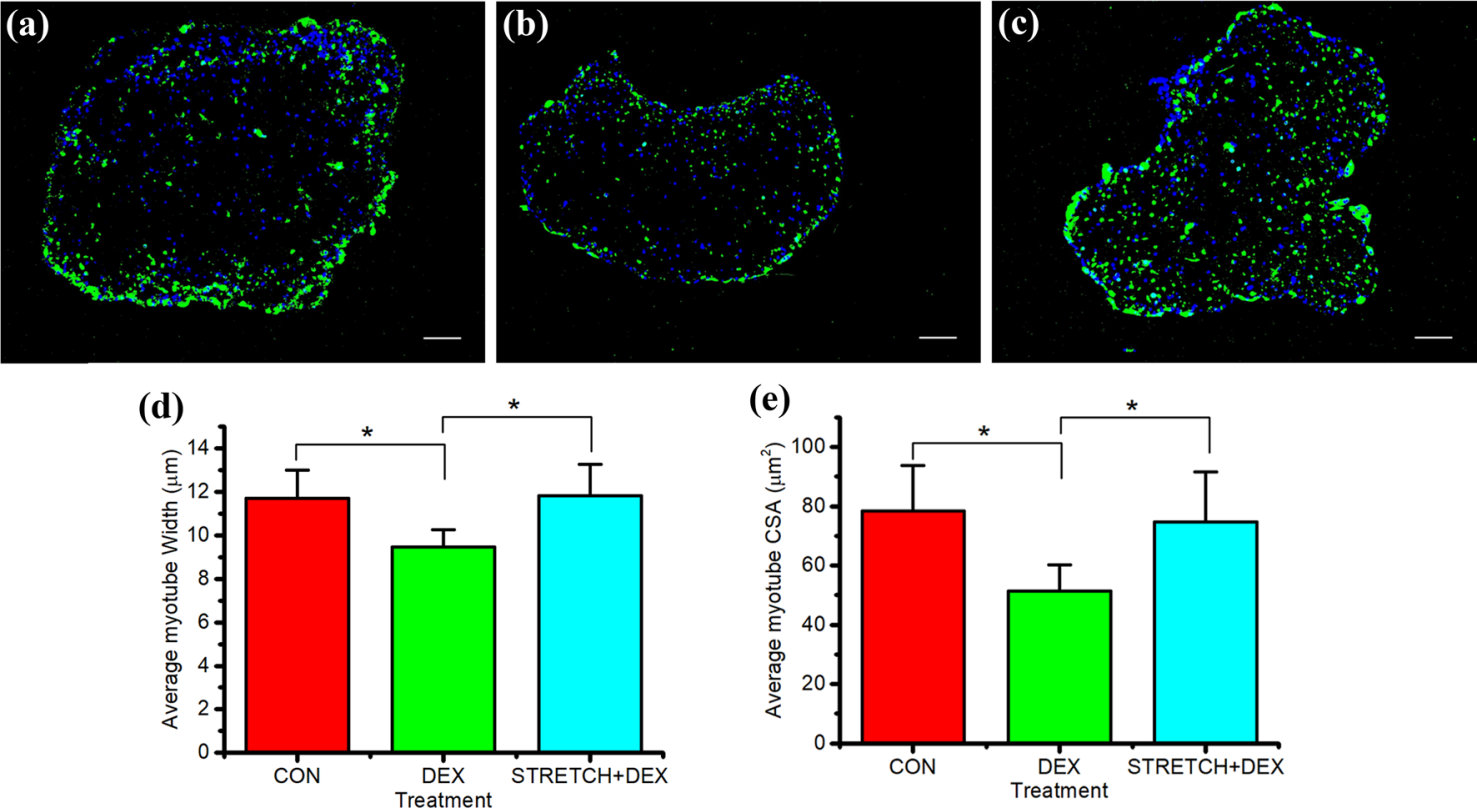
Immunohistochemical fluorescent staining of the nucleic DNA (blue) and muscle specific protein filament MyHC (green) in cross sections of engineered muscles (× 10 magnification) **a** representative CON (no DEX administration at day 14), **b** 40 µM DEX administration over 24 h (DEX), **c** Mechanical load for 3 h following by 40 µM DEX administration over 24 h (STRETCH + DEX), **d** average myotube width (µm), **e** average cross sectional area (CSA) of the myotubes (μm^2^) of CON (Control at day 14), DEX and STRETCH + DEX. Scale bar represents 100 μm. Significantly different values are identified using*. All data presented as mean ± SD, from n = 6 engineered muscles, obtained from three independent experiments. (Color figure online)

Furthermore, average myotube width and CSA were measured after 3 h of mechanical loading followed by 24 h of DEX administration (Fig. 5). Preconditioning the engineered muscles with mechanical load prior to 24 h of DEX treatment prevented the reduction in average myotube width and CSA observed when DEX was administered alone (P < 0.05). Furthermore, when comparing CON and preconditioned engineered muscle after DEX treatment, no significant differences were observed in average myotube width and CSA (P = 0.879 and 0.641 respectively). Therefore, a bout of mechanical loading prior to DEX administration has a protective effect on myotube atrophy.

### Mechanical loading positively effects maximal force production in engineered muscles treated with DEX

**Fig. 6:**
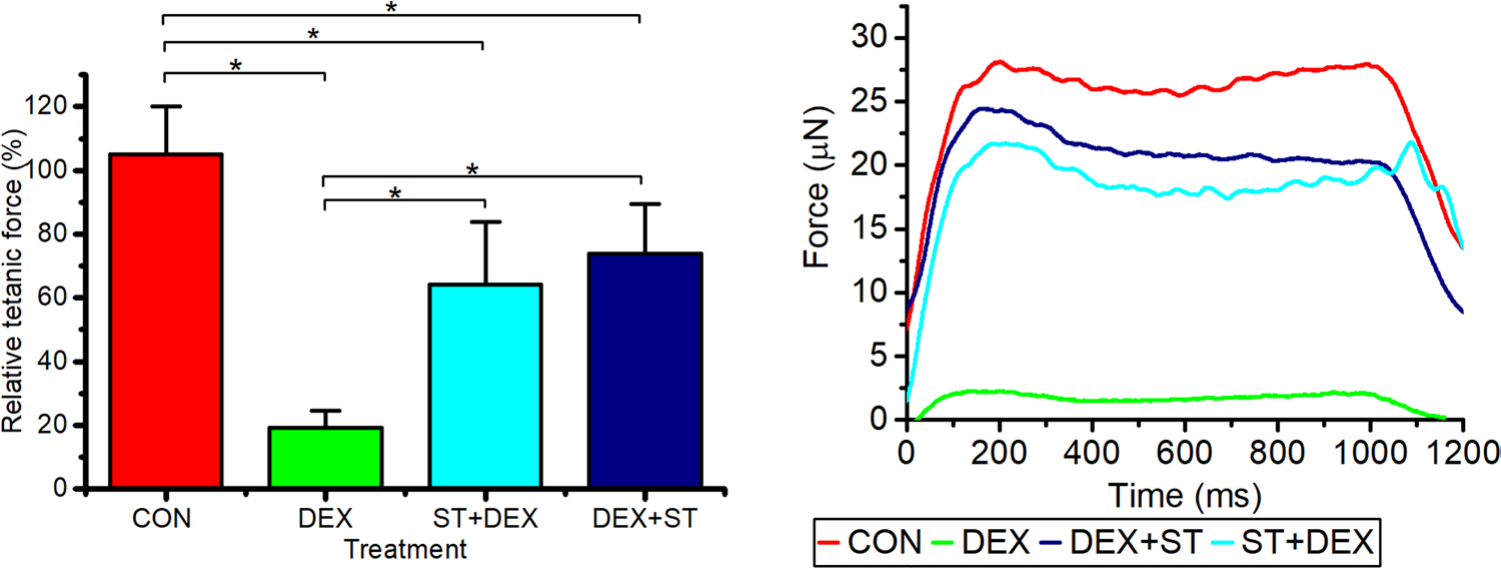
Maximal contractile tetanic force of engineered skeletal muscle at CON (control day 14, n = 6), 40 µM DEX administration over 24 h (DEX, n = 10), mechanically loaded for 3 h then administered 40 µM DEX over 24 h (STRETCH + DEX, n = 9) and 40 µM DEX administration over 24 h replaced with differentiation media and mechanically loaded for 3 h and sampled after 48 h (DEX + STRETCH, n = 6). All cultures were compared to CON at day 14 within individual experimental repeats to calculate relative tetanic force. All data presented as mean ± SD, obtained from three independent experiments

Finally, maximal contractile force of the engineered muscle was measured in order to verify whether the contractility of engineered muscles was successfully recovered through mechanical loading following DEX treatment and also whether mechanical loading prior to DEX treatment had a positive effect on contractility (Fig. 6). 24 h of DEX treatment induced a significant reduction (~ 85%) in maximal force production (p < 0.001), which was increased (between ~ 45 and 55%) through mechanical loading both prior to and post DEX treatment, when compared to DEX alone (p < 0.001). No significant advantage was obtained when comparing mechanical loading prior to or post DEX treatment (P = 0.212). Thus, mechanical loading successfully protected and reversed functional decrements within the engineered muscles following a bout of DEX-induced atrophy.

## Discussion

Skeletal muscle atrophy as a consequence of disease or disuse is associated with increased morbidity and mortality (Powers et al. 2016), and as such interventions aimed at preventing and/or reversing atrophy have great clinical importance. In the present study we mechanically loaded 3D tissue engineered skeletal muscle either prior or subsequent to treatment with 40 µM DEX and found that loading can offset the induction of catabolic genes associated with DEX treatment, as well as prevent atrophy and decrements in tissue contractile function.

Previously DEX has successfully induced skeletal muscle atrophy in vivo (Fappi et al. 2019), and 24 h DEX exposure can induce atrophy and loss of muscle function in vitro (Barassi et al. 2016; Castillero et al. 2013; Shimizu et al. 2017)*.* In our system 40 µM of DEX was the lowest concentration that induced significant atrophy and impaired muscle function, and is comparable to concentrations previously used in a fibrin/Matrigel engineered skeletal muscle system to induce similar rapid decreases in functionality (Shimizu et al. 2017)*.* Although we found further decrements in engineered muscle function at higher DEX concentrations, it has been previously reported that doses of DEX greater than 100 µM administered over extended periods (> 24 h) can significantly impair the contractile capability and sarcomere structures of the myotubes present within the engineered muscles, rendering the engineered muscles almost completely functionless after 48 h (Shimizu et al. 2017), and as such we chose to use 40 µM of DEX for 24 h in the remainder of our experiments.

We have recently shown that mechanically loading 3D tissue engineered skeletal muscle for 3 h induces anabolic signalling and hypertrophy (Aguilar-Agon et al. 2019). In the present study we found that this same loading stimulus applied following 24 h of DEX treatment can offset the induction of MuRF-1 and MAFbx mRNA. Similarly, primary human muscle contraction performed immediately following immobilization (a potent atrophic stimulus) quickly activates alterations in gene expression associated with the suppression of muscle catabolism. This leads to an induction in skeletal muscle hypertrophy and remodelling, specifically indicating a decline in MAFbx and MuRF-1 (Jones et al. 2004). It is well recognised that MAFbx and MuRF-1 are critical regulators of muscle atrophy through the UPP (Gomes et al. 2001; Lecker et al. 2004). MuRF-1 has been shown to preferentially interact with structural proteins such as titin, degrading myosin heavy chain (MyHC) and thus control protein degradation following DEX administration (Centner et al. 2001). Given the crucial roles of MuRF-1 and MAFbx in the regulation of muscle protein degradation, it is unsurprising that we also observed myotube atrophy following DEX administration. Positively, mechanical loading of the engineered muscles following DEX administration produced a significant improvement in average myotube width and CSA when compared to the administration of DEX alone. This finding is consistent with the reported reversal of suppressed MyHC synthesis following in vitro mechanical load of DEX treated muscle cells (Chromiak and Vandenburgh 1992). Thus, the confirmed recovery in myotube size following DEX administration we hypothesize was due to a reduction in catabolic gene expression, nevertheless the anabolic effects of exercise may also aid myotube hypertrophy. These findings further confirm the physiological accuracy of our engineered muscles as a model system for skeletal muscle adaptation, which can be utilized to aid our understanding on the underlying pathophysiology and treatment options associated with skeletal muscle wasting.

It has been demonstrated in rodents that loading of a muscle prior to immobilisation affords a protective influence on muscle mass (Petrini et al. 2016; Sun et al. 2013). We next investigated this possibility in our model system by loading the muscle for 3 h prior to 24 h of DEX treatment. Indeed, we found that loading prevented the decline in myotube size induced by DEX treatment alone. Furthermore, prior loading of the muscle blocked the characteristic increase in the mRNA levels of both MuRF-1 and MAFbx, therefore offering a potential mechanism to explain this protective effect. Interestingly, previously it has been observed that in MuRF-1 knockout mice muscle mass was spared following 14 days of DEX treatment, whereas this protective effect was not afforded to MAFbx knockout mice (Baehr et al. 2011). Thus, although we saw an attenuation of both MAFbx and MuRF-1 mRNA when mechanical loading preceded DEX treatment, we can speculate that the protective effect on myotube size is mediated largely through MuRF-1. Since we solely measured the mRNA levels of ubiquitin ligases MuRF-1 and MAFbx in our experiments and not their activity at a protein level, we cannot discount the role of other potential mediators of muscle mass in explaining the preservation of myotube size. For example, a large body of evidence suggests that myostatin is an important regulator of skeletal muscle mass (e.g. Taylor et al. 2001; Thomas et al. 2000) and numerous other intracellular mediators such as FOXO, GSK3ß, p300, REDD1 and ATF4 are also involved in skeletal muscles catabolic and anti-anabolic effects of GC (Schakman et al. 2008). Further research into these other intracellular mediators would be advantageous when examining the role of mechanical load on DEX-induced skeletal muscle atrophy. Engineered skeletal muscle provides a unique model to mechanistically interrogate these pathways, alongside much needed human trials to explore whether pre-conditioning skeletal muscle prior to an atrophic can have similar protective effects on muscle mass to those described herein.

Finally, we tested whether mechanical loading either prior- or subsequent to DEX treatment, could preserve maximal force production. Indeed, muscle functionality is a clinically important predictor of mortality under periods of atrophy (Manini et al. 2007). We were able to establish improved functionality of the engineered muscle subject to mechanical load following DEX treatment, through a ~ fourfold increase in maximal contractile force production compared to engineered muscle treated with 24 h DEX. Furthermore, the positive effects of preconditioning prior to DEX-induced atrophy and improved functionality were highlighted showing a ~ threefold increase in maximal force production, similar to that seen following DEX treatment and also the addition of IGF-1 to DEX treated engineered muscles (Shimizu et al. 2017). Interestingly, increased performance and maximal force output can be also observed in humans following strength training during simulated space travel (Alkner et al. 2003), further highlighting the protective effects of muscle loading not only prior to or subsequent to a bout of atrophy, however also during atrophy inducing conditions such as space flight.

In summary, this study shows for the first time that mechanical loading of in vitro 3D engineered muscle can offset both atrophy and functional impairments associated with DEX treatment, regardless of whether the loading occurs before or after DEX treatment. Furthermore, we have identified that mechanical loading of the muscle can prevent increases in MuRF-1 and MAFbx mRNA expression which may provide an indication as to the molecular mechanism underpinning the observed physiological effects. Moreover, this study demonstrates the application of tissue engineered muscle to the study of skeletal muscle health and disease and offers great potential for future use to better understand treatment modalities for skeletal muscle atrophy, within a highly controlled and high throughput environment.

## Electronic supplementary material

Below is the link to the electronic supplementary material.

Supplementary file1 (DOCX 20 kb)

## Data Availability

All the data supporting the findings of this study are available within the paper and its supplementary material.
