# Supplementary material for: Mechanical loading of tissue engineered skeletal muscle prevents dexamethasone induced myotube atrophy
Source: J Muscle Res Cell Motil. 2020 Sep 21;42(2):149–59. doi: 10.1007/s10974-020-09589-0 (PMC8332579; doi:10.1007/s10974-020-09589-0)
Supplement: Supplementary file 1 — Supplementary file1 (DOCX 20 kb) [file 10974_2020_9589_MOESM1_ESM.docx]

**Mechanical loading of tissue engineered skeletal muscle prevents dexamethasone induced myotube atrophy**

Kathryn W. Aguilar-Agon^1^, Andrew J. Capel^1^, Jacob W. Fleming^1^, Darren J. Player^2^, Neil R.W. Martin^1^and Mark P. Lewis^1*^

**Supplementary material**

The following annotated text describes the application of the in-house macro described within the methods (2.5 Histology). Text in red is descriptive annotation and not part of the macro.

**MyHC % Calculation Macro**

run("Clear Results");

roiManager("Reset");

title=getTitle();

// Clears existing data from prior runs

delimiters =".";

stringResult = split(title, delimiters);

title2=stringResult[0];

selectWindow(title);

s=nSlices();

run("Set Scale...", "distance=1392 known=1283 pixel=1 unit=um");

// Sets scale to 10x magnification.

run("Duplicate...", "title="+title2+"_mask duplicate range=1-"+s+"");

// Duplicates the image file.

run("8-bit");

// Converts image file to 8 bits.

run("Despeckle");

run("Smooth");

// De-speckles and smooths image to remove noise.

run("adaptiveThr ");

// Runs adaptive threshold macro (see below).

run("Set Measurements...", "area ellipse feret's stack redirect="+title2+"_mask decimal=3");

// Defines measurements.

run("Invert", "stack");

// Inverts image for analysis.

Answer=getBoolean("Apply Watershed?");

if (Answer==true) run("Watershed", "stack");

// Applies the watershed feature to separate features.

run("Analyze Particles...", "size=25-50000000 show=Outlines display exclude add stack");

// Analyses particles for defined size range.

showStatus ("Done");

// Completes analysis and displays data.

The threshold macro utilised above is available for download free of charge from imagej.net.
